# Open Access and Article Processing Charges in Cardiology and Cardiac Surgery Journals: a Cross-Sectional Analysis

**DOI:** 10.21470/1678-9741-2021-0289

**Published:** 2021

**Authors:** Dominique Vervoort, Jessica G.Y. Luc, Michel Pompeu B. O. Sá, Eric W. Etchill

**Affiliations:** 1 Department of Health Policy and Management, Johns Hopkins Bloomberg School of Public Health, Baltimore, Maryland, United States of America.; 2 Division of Cardiovascular Surgery, Department of Surgery, University of British Columbia, Vancouver, British Columbia, Canada.; 3 Division of Cardiovascular Surgery, Pronto-Socorro Cardiológico de Pernambuco (PROCAPE), Universidade de Pernambuco, Recife, Pernambuco, Brazil.; 4 Department of Surgery, Johns Hopkins University School of Medicine, Baltimore, Maryland, United States of America.

**Keywords:** Open Access Publishing, Access to Information, Publishing, Cardiac Surgical Procedures, Cardiology, Periodicals as Topic

## Abstract

**Introduction:**

Open access (OA) publishing often requires article processing charges (APCs). While OA provides opportunities for broader readership, authors able to afford APCs are more commonly associated with well-funded, high-income country institutions, skewing knowledge dissemination. Here, we evaluate publishing models, OA practices, and APCs in cardiology and cardiac surgery.

**Methods:**

The InCites Journal Citation Reports 2019 directory by Clarivate Analytics was searched for “Cardiac and Cardiovascular Systems” journals. Sister journals of included journals were identified. All journals were categorized as predominantly cardiology or cardiac surgery. Publishing models, APCs, and APC waivers were defined for all journals.

**Results:**

One hundred sixty-one journals were identified (139 cardiology, 22 cardiac surgery). APCs ranged from $244 to $5,000 ($244-5,000 cardiology; $383-3,300 cardiac surgery), with mean $2,911±891 and median $3,000 (interquartile range [IQR]: $2,500-3,425) across 139 journals with non-zero available APCs ($2,970±890, median $3,000, IQR: $2,573-3,450, cardiology; $2,491±799, median $2,740, IQR: $2,300-3,000, cardiac surgery). Average APCs were $3,307±566 and median $3,250 (IQR: $3,000-3,500) for hybrid journals ($3,344±583, median $3,260, IQR: $3,000-3,690, cardiology; $2,983±221, median $2,975, IQR: $2,780-3,149, cardiac surgery) and $1,997±832 and median $2,100 (IQR: $1,404-2,538) for fully OA journals ($2,039±843, median $2,100, IQR: $1,419-2,604, cardiology; $1,788±805, median $2,000, IQR: $1,475-2,345, cardiac surgery). Waivers were available for 51 (86.4%) fully OA and 37 (37.4%) hybrid journals. Seventeen journals were fully OA without APCs, one journal did not yet release APCs, and four journals were subscription-only.

**Conclusion:**

OA publishing is common in cardiology and cardiac surgery with substantial APCs. Waivers remain limited, posing barriers for unfunded and lesser-funded researchers.

**Table:** 

Abbreviations, acronyms & symbols				
APCs	= Article processing charges		JAMA	= Journal of the American Medical Association
BMC	= BioMed Central		JCR	= Journal Citation Reports
EP Europace	= The European Journal of Pacing, Arrhythmias and Cardiac Electrophysiology		JTCVS	= Journal of Thoracic and Cardiovascular Surgery
ESC	= European Society of Cardiology		OA	= Open access
IQR	= Interquartile range		REC	= Revista Española de Cardiología
JACC	= Journal of the American College of Cardiology		USD	= U.S. Dollar

## INTRODUCTION

Scientific publishing is a multibillion-dollar industry, costing steep license fees for institutions to provide access to journals, high individual costs to subscribe to journals or buy articles, and little to no remuneration for reviewers’ time. Authors not associated with institutions covering full or partial access to major journals are forced to find alternate methods of accessing publications, such as pirating, especially if unable to afford fees to access articles^(1)^. Similarly, authors are frequently charged article processing charges (APCs) to publish “open access” (OA). This further impedes early-career researchers, researchers from lesser-funded institutions, and researchers from most low- and middle-income countries to publish articles^(2)^. In response to high publishing fees, predatory journals are increasingly pervading scientific practice^(3)^. Predatory journals promise quick and easy OA publishing for a fee, typically at only a fraction of non-predatory journals, resulting in millions of dollars generated by these journals, even during the coronavirus disease 2019 (or COVID-19) pandemic^(4)^. 

Despite the growing OA discourse, little is known regarding the most common subscription models and APCs. This study evaluates publishing models, OA practices, and APC amounts in cardiology and cardiac surgery journals.

## METHODS

The InCites Journal Citation Reports (JCR) directory by Clarivate Analytics was searched to identify journals categorized as “Cardiac and Cardiovascular Systems” for 2019. Sister journals of included journals, defined as journals published by the same publisher and associated with the included journal, were further included. Journals were manually distinguished as being predominantly related to cardiology or to cardiac surgery based on their titles and, where applicable, associated professional societies. Publishing models (OA only, subscription only, or hybrid) and APCs, if applicable, were defined for all journals. OA only was defined as journals making all articles publicly available to readers, subscription only as journals making articles only available to readers with a (personal or institutional) subscription, and hybrid as a combination thereof. APCs were presented as mean±standard deviation and median with interquartile ranges (IQR). For journals with APCs, the availability of partial or full waivers as presented on the journal’s or publisher’s website was recorded.

## RESULTS

In 2019, 137 non-duplicate journals were identified in the JCR Cardiac and Cardiovascular Systems category. Twenty-four journals were identified as sister journals of JCR-indexed journals for a total of 161 journals. One hundred thirty-nine journals were categorized as predominantly cardiology, of which 88 were hybrid, 49 fully OA, and two subscription. Twenty-two journals were categorized as predominantly cardiac surgery, of which 10 were hybrid, 10 fully OA, and two subscription.

APCs ranged from $244 to $5,000 ($244-5,000 cardiology; $383-3,300 cardiac surgery), with an average of $2,911±891 and median of $3,000 (IQR: $2,500-3,425) across 139 journals with non-zero and available APCs ($2,970±890, median $3,000, IQR: $2,573-3,450, cardiology; $2,491±799, median $2,740, IQR: $2,300-3,000, cardiac surgery). Average APCs were $3,307±566 and median $3,250 (IQR: $3,000-3,500) for hybrid journals ($3,344±583, median $3,260, IQR: $3,000-3,690, cardiology; $2,983±221, median $2,975, IQR: $2,780-3,149, cardiac surgery) and $1,997±832 and median $2,100 (IQR: $1,404-2,538) for fully OA journals ($2,039±843, median $2,100, IQR: $1,419-2,604, cardiology; $1,788±805, median $2,000, IQR: $1,475-2,345, cardiac surgery). Waivers were available for 51 (86.4%) fully OA journals, of which 16 (14 cardiology; two cardiac surgery) automatically applied waivers for authors from eligible low- and middle-income countries. Waivers were available for 37 (37.4%) hybrid journals, none of which were automatically applied. Seventeen journals (14 cardiology; three cardiac surgery) were fully OA and did not charge APCs. Supplemental Tables 1 and 2 present findings for cardiology and cardiac surgery journals. All values are presented in U.S. Dollars.

**Supplemental Table 1:** Publishing models and article processing charges for cardiology journals indexed in InCites Journal Citation Reports.

Journal Name	Impact Factor	Publishing Model	Article Processing Charges (USD)	Waivers Available
Acta Cardiologica	1.208	Hybrid	2,995	No
American Heart Journal	4.153	Hybrid	3,250	Case by case (Research4Life)
American Journal of Cardiology	2.570	Hybrid	2,960	Case by case (Research4Life)
American Journal of Cardiovascular Drugs	2.674	Hybrid	3,860	No
American Journal of Physiology: Heart and Circulatory Physiology	3.864	Hybrid	3,000	No
Anatolian Journal of Cardiology	1.223	Open Access	Free	-
Annals of Noninvasive Electrocardiology	1.131	Open Access	2,250	Yes, upon request
Annals of Thoracic Medicine	1.456	Open Access	Free	-
Archives of Cardiovascular Diseases	2.434	Hybrid	3,620	Case by case (Research4Life)
Arquivos de Brasileiros de Cardiologia	1.450	Open Access	Free	-
Atherosclerosis	3.919	Hybrid	3,200	Case by case (Research4Life)
Basic Research in Cardiology	11.981	Hybrid	4,390	No
BMC Cardiovascular Disorders	2.078	Open Access	2,380	Case by case
Canadian Journal of Cardiology	5.234	Hybrid	3,200	Case by case (Research4Life)
Canadian Journal of Cardiology Open	-	Open Access	2,100	Automatic (Research4Life)
Cardiology	1.791	Hybrid	3,530	No
Cardiology Clinics	1.811	Hybrid	3,150	Case by case (Research4Life)
Cardiology in Review	1.816	Hybrid	2,400	No
Cardiology in the Young	1.000	Hybrid	3,160	No
Cardiology Journal	1.669	Open Access	900	Case by case
Cardiology Research and Practice	1.292	Open Access	2,100	Automatic
Cardiorenal Medicine	1.754	Open Access	2,580	Case by case
Cardiovascular and Interventional Radiology	2.034	Hybrid	3,860	No
Cardiovascular Diabetology	7.332	Open Access	3,170	Case by case
Cardiovascular Diagnosis and Therapy	2.615	Open Access	1,390	Yes, upon request
Cardiovascular Digital Health Journal	-	Open Access	2,800	Automatic (Research4Life)
Cardiovascular Drugs and Therapy	4.069	Hybrid	3,860	No
Cardiovascular Engineering and Technology	1.771	Hybrid	3,260	No
Cardiovascular Journal of Africa	0.897	Open Access	461	No
Cardiovascular Pathology	1.756	Hybrid	3,000	Case by case (Research4Life)
Cardiovascular Research	8.168	Hybrid	4,592	No
Cardiovascular Therapeutics	2.538	Open Access	2,100	Automatic
Cardiovascular Toxicology	2.284	Hybrid	3,860	No
Cardiovascular Ultrasound	2.051	Open Access	2,790	Case by case
Catheterization and Cardiovascular Interventions	2.044	Hybrid	3,800	No
Circulation	23.603	Hybrid	3,450	No
Circulation: Arrhythmia and Electrophysiology	4.393	Hybrid	3,450	No
Circulation: Cardiovascular Imaging	5.691	Hybrid	3,450	No
Circulation: Cardiovascular Interventions	5.493	Hybrid	3,450	No
Circulation: Cardiovascular Quality & Outcomes	5.071	Hybrid	3,450	No
Circulation: Genomic and Precision Medicine	4.534	Hybrid	3,450	No
Circulation: Heart Failure	6.033	Hybrid	3,450	No
Circulation Research	14.467	Hybrid	3,450	No
Circulation Journal	2.540	Open Access	1,929	No
Clinical Cardiology	2.248	Open Access	2,475	Yes, upon request
Clinical Research in Cardiology	5.268	Hybrid	3,760	No
Clinical Research in Cardiology Supplements	-	Hybrid	2,760	No
Congenital Heart Disease	1.663	Open Access	1,200	Case by case
Coronary Artery Disease	1.335	Hybrid	2,400	No
Current Cardiology Reports	2.434	Hybrid	3,860	No
Current Opinion in Cardiology	2.149	Hybrid	3,000	No
Current Problems in Cardiology	2.966	Hybrid	3,000	Case by case (Research4Life)
e-Journal of Cardiology Practice	-	Open Access	Free (invited submissions only)	-
Echocardiography	1.393	Hybrid	2,700	No
ESC Heart Failure	3.902	Open Access	3,366	Case by case (Research4Life)
EuroIntervention	3.993	Open Access	Free	-
EP Europace	4.045	Hybrid	4,592	No
European Heart Journal	22.673	Hybrid	4,592	No
European Heart Journal: Acute Cardiovascular Care	3.813	Hybrid	3,300	No
European Heart Journal: Cardiovascular Imaging	4.841	Hybrid	4,549	No
European Heart Journal: Cardiovascular Pharmacotherapy	6.696	Hybrid	3,830	No
European Heart Journal: Case Reports	-	Open Access	726	Yes, upon request
European Heart Journal: Digital Health	-	Open Access	3,225	Yes, upon request
European Heart Journal: Quality of Care and Clinical Outcomes	-	Hybrid	4,280	No
European Heart Journal Supplements	1.655	Hybrid	4,592	No
European Journal of Cardiovascular Nursing	2.296	Hybrid	3,300	No
European Journal of Heart Failure	11.627	Hybrid	4,300	No
European Journal of Preventive Cardiology	5.864	Hybrid	3,300	No
Frontiers in Cardiovascular Medicine	3.915	Open Access	2,490	Case by case
Global Heart	3.862	Open Access	1,583	Automatic discount
Heart	5.213	Hybrid	3,100	No
Heart and Vessels	1.618	Hybrid	3,260	No
Heart Failure Clinics	2.327	Hybrid	2,680	Case by case (Research4Life)
Heart Failure Reviews	3.538	Hybrid	3,860	No
Heart & Lung	1.630	Hybrid	2,860	Case by case (Research4Life)
Heart, Lung and Circulation	2.194	Hybrid	2,500	Case by case (Research4Life)
Heart Rhythm	5.731	Hybrid	3,500	Case by case (Research4Life)
Heart Rhythm: Case Reports	-	Open Access	1,050	Automatic (Research4Life)
Heart Rhythm O2	-	Open Access	2,150	Automatic (Research4Life)
Hellenic Journal of Cardiology	4.047	Open Access	Free	-
Herz	1.033	Hybrid	2,760	No
International Heart Journal	1.906	Open Access	1,447	No
International Journal of Cardiology	3.229	Hybrid	3,470	Case by case (Research4Life)
International Journal of Cardiology: Congenital Heart Disease	-	Open Access	2,000	Automatic (Research4Life)
International Journal of Cardiology: Heart and Vasculature	-	Open Access	2,550	Automatic (Research4Life)
International Journal of Cardiovascular Imaging	1.969	Hybrid	3,260	No
JAMA Cardiology	12.794	Hybrid	5,000	No
Journal of Cardiology	2.246	Hybrid	3,000	Case by case (Research4Life)
Journal of Cardiopulmonary Rehabilitation and Prevention	1.383	Hybrid	3,375	No
Journal of Cardiovascular Computed Tomography	2.892	Hybrid	3,150	Case by case (Research4Life)
Journal of Cardiovascular Electrophysiology	2.424	Hybrid	3,900	No
Journal of Cardiovascular Magnetic Resonance	5.361	Open Access	2,680	Case by case
Journal of Cardiovascular Medicine	1.225	Hybrid	2,800	No
Journal of Cardiovascular Nursing	1.675	Hybrid	2,570	No
Journal of Cardiovascular Pharmacology	2.598	Hybrid	3,150	No
Journal of Cardiovascular Pharmacology and Therapeutics	2.322	Hybrid	3,000	No
Journal of Cardiovascular Translational Research	3.312	Hybrid	3,860	No
Journal of Electrocardiology	0.944	Hybrid	2,830	Case by case (Research4Life)
Journal of Geriatric Cardiology	2.491	Open Access	Free	-
Journal of Interventional Cardiac Eletrophysiology	1.277	Hybrid	3,260	No
Journal of Interventional Cardiology	1.758	Open Access	2,100	Automatic
Journal of Invasive Cardiology	1.470	Open Access	Free	-
Journal of Molecular and Cellular Cardiology	4.133	Hybrid	3,320	Case by case (Research4Life)
Journal of Nuclear Cardiology	3.366	Hybrid	3,860	No
Journal of the American Heart Association	4.605	Open Access	2,750	Automatic
Journal of the American College of Cardiology (JACC)	20.589	Hybrid	3,000	Case by case (Research4Life)
JACC: Basic to Translational Science	-	Open Access	3,400	Automatic (Research4Life)
JACC: CardioOncology	-	Open Access	2,500	Automatic (Research4Life)
JACC: Cardiovascular Imaging	12.740	Hybrid	3,000	Case by case (Research4Life)
JACC: Cardiovascular Interventions	8.432	Hybrid	3,000	Case by case (Research4Life)
JACC: Case Reports	-	Open Access	600	Automatic (Research4Life)
JACC: Clinical Electrophysiology	-	Hybrid	3,000	Case by case (Research4Life)
JACC Heart Failure	9.140	Hybrid	2,500	Case by case (Research4Life)
Journal of Cardiac Failure	3.623	Hybrid	3,040	Case by case (Research4Life)
Journal of the American Society of Echocardiography	5.508	Hybrid	3,000	Case by case (Research4Life)
Journal of Thrombosis and Thrombolysis	2.054	Hybrid	3,860	No
Kardiologia Polska	1.874	Open Access	Free	-
Kardiologiya	0.264	Subscription	-	-
Korean Circulation Journal	2.322	Open Access	Free	-
Minerva Cardioangiologica	0.713	Subscription	-	-
Nature Reviews Cardiology	20.260	Hybrid (as of 2021)	Not yet available	No
Netherlands Heart Journal	1.933	Open Access	Free	-
Nutrition Metabolism and Cardiovascular Diseases	3.700	Hybrid	3,000	Case by case (Research4Life)
Open Heart	-	Open Access	2,628	Case by case
Pace-Pacing and Clinical Electrophysiology	1.303	Hybrid	3,200	No
Pediatric Cardiology	1.564	Hybrid	3,260	No
Perfusion-United Kingdom	1.234	Hybrid	3,000	No
Postepy w Kardiologii Interwencyjnej	1.347	Open Access	244	No
Progress in Cardiovascular Diseases	6.763	Hybrid	2,800	Case by case (Research4Life)
Pulmonary Circulation	2.205	Open Access	2,000	Case by case
Respiratory Medicine	3.095	Hybrid	3,500	Case by case (Research4Life)
Reviews in Cardiovascular Medicine	0.659	Open Access	1,250	Case by case
Revista Española de Cardiología (REC)	4.642	Hybrid	3,000	Case by case (Research4Life)
REC: CardioClinics	-	Hybrid	1,500	Case by case (Research4Life)
REC: Interventional Cardiology	-	Open Access	Free	-
Revista Portuguesa de Cardiologia	0.960	Open Access	Free	-
Scandinavian Cardiovascular Journal	1.084	Hybrid	2,995	No
Texas Heart Institute Journal	1.023	Open Access	Free	-
Trends in Cardiovascular Medicine	4.755	Hybrid	3,300	Case by case (Research4Life)

BMC=BioMed Central; EP Europace=The European Journal of Pacing, Arrhythmias and Cardiac Electrophysiology; ESC=European Society of Cardiology; JAMA=Journal of the American Medical Association; USD=U.S. Dollar

**Supplemental Table 2:** Publishing models and article processing charges for cardiac surgery journals indexed in InCites Journal Citation Reports.

Journal Name	Impact Factor	Publishing Model	Article Processing Charges (USD)	Waivers Available
Annals of Cardiothoracic Surgery	3.058	Open access	2,390	No
Annals of Thoracic and Cardiovascular Surgery	1.584	Open access	383	No
Annals of Thoracic Surgery	3.639	Hybrid	3,000	Case by case (Research4Life)
Brazilian Journal of Cardiovascular Surgery	1.053	Open access	Free	-
European Journal for Cardio-Thoracic Surgery	3.486	Hybrid	3,087	No
General Thoracic and Cardiovascular Surgery	1.088	Hybrid	3,260	No
Heart Surgery Forum	0.404	Open Access	950	No
Interactive CardioVascular and Thoracic Surgery	1.675	Open Access	Free	-
Journal of Cardiac Surgery	1.490	Hybrid	2,900	No
Journal of Cardiothoracic and Vascular Anesthesia	2.258	Hybrid	2,680	Case by case (Research4Life)
Journal of Cardiothoracic Surgery	1.506	Open Access	2,490	Case by case
Journal of Cardiovascular Surgery	1.415	Subscription	-	-
Journal of Heart and Lung Transplantation	7.865	Hybrid	3,300	Case by case (Research4Life)
Journal of Thoracic and Cardiovascular Surgery (JTCVS)	4.451	Hybrid	2,950	Case by case (Research4Life)
JTCVS Open	-	Open Access	2,000	Automatic (Research4Life)
JTCVS Techniques	-	Open Access	2,000	Automatic (Research4Life)
Multimedia Manual of Cardio-Thoracic Surgery	-	Open Access	Free	-
Operative Techniques in Thoracic and Cardiovascular Surgery	-	Hybrid	2,740	Case by case (Research4Life)
Seminars in Thoracic and Cardiovascular Surgery	2.133	Hybrid	2,740	Case by case (Research4Life)
Seminars in Thoracic and Cardiovascular Surgery: Pediatric Cardiac Surgery Annual	-	Hybrid	3,170	Case by case (Research4Life)
Thoracic and Cardiovascular Surgeon	1.209	Subscription	-	-
Thoracic and Cardiovascular Surgeon Reports	-	Open Access	2,300	No

USD=U.S. Dollar

## DISCUSSION

OA publishing is widely available as hybrid or fully OA journals in cardiology and cardiac surgery, yet APCs vary from $244 to $5,000 per article. APC waivers were available for authors from eligible low- and middle-income countries, either automatically applied or on a case-by-case basis. Few journals considered financial need among authors from high-income countries, presumably with the assumption that authors would be able to find the means to cover the costs. Our findings suggest that, while the academic shift to OA is clearly visible^(5)^, enormous financial barriers on non-funded authors remain in the pursuit of OA publishing regardless of home country or institution. Although research on OA and APCs remains limited, lower average APCs were found in ophthalmology, multiple sclerosis research, and global health^(6-8)^.

While waivers were often considered by publishers, these were typically only considered when publishing in a fully OA journal. Most journals publishing as hybrid models did not have waiver availability, requiring authors to opt for subscription publication. Hybrid journals were on average $1,300 more expensive than fully OA journals, which is confirmed by previous findings from research spending by North American institutions^(9)^. As these journals make up a majority of journals, especially those with higher impact factor, few authors are able to seek OA publishing of their work without having research funding or going through tedious processes of obtaining one-off external funding. Furthermore, while a majority of publishers clearly listed APCs, some publishers did not make APCs publicly available, which may deter and even deceive authors. Moreover, amongst the available waivers and discount criteria, researchers from upper-middle-income countries find themselves in an “upper-middle-income trap”, as they are commonly not eligible for such discounts or waivers despite lower income and standards of living compared to high-income countries. For example, authors from Brazil would not be eligible for discounts or waivers amongst the majority of main publishers (and thus journals); yet, there is vast variation in institutional and personal funding availability, with most researchers being unable to bear APCs. Lastly, APC waivers were typically defined on the basis of authors’ affiliation, which may limit authors from low- and middle-income countries who have recently moved to or are studying or training in high-income countries, authors from lower socioeconomic status, or authors without research funding (*e.g*., non-doctoral graduate students, residents) whose institution is not based in eligible countries. Some journals offer minimal discounts (*e.g*., 10-15% discount) to partnering institutions, which may perpetuate barriers for those without funding and only skew the dissemination of research towards those with research funding. While some smaller journals are independently operating or associated with non-profit organizations or professional societies, most journal have contracts with large publishing entities that jointly comprise the billion-dollar academic publishing industry, wherein profit margins run up to 30%^(2)^. Few OA or hybrid journals had no APCs and were most commonly covering or based in Latin America, where free-of-charge OA journals are commonplace and supported by both professional society and governmental support, a model that may be extended elsewhere^(10)^.

Publishing does not come without a cost, as journals and publishers must hire editorial staff, manage online platforms and servers, maintain publication relations, and ensure typesetting of accepted publications. Nevertheless, they equally rely on the voluntary time of reviewers and most editors, whereas societal, industry, and other partnerships typically result in substantial monetary and in-kind support for journals to cover large shares of the fixed costs. The true costs of publishing a single article remain unclear, but estimates have shown to be as low as US$300 per article, far lower than the requested APCs^(11)^.

The commonly cited publish-or-perish mentality in academic medicine has led to an increase in the number of predatory journals, seeking to take advantage of vulnerable authors facing barriers to publishing in acknowledged journals or wanting to find a quick way to publish. Previously known cardiovascular journals have been bought to leverage a pre-existing reputation and attract clinicians and researchers, yet they only publish verbatim (*i.e*., not reviewing or editing submissions), generating tens up to hundreds of thousands of dollars per journal per year^(12)^. What is especially concerning is the potentially important knowledge remaining undetected and not disseminated as a result of such predatory journals. For example, the field of global cardiac surgery is nascent, and local studies reporting on cardiac surgery outcomes in low- and middle-income countries have been identified in predatory journals. Barriers for authors from these countries are upheld by high APCs (less able to publish in certain journals) and non-OA (less able to access articles), underscoring the need to change the academic publishing narrative.

### Limitations

Several limitations are inherent to this analysis. We used JCR to identify established journals and their sister journals, but we are aware of a number of journals not listed on JCR that are legitimate *(e.g*., PubMed/MEDLINE indexing, societal affiliation). However, as the number of journals included on JCR is large, our analysis paints a representative picture. Moreover, various journals give discounts for society members or on a case-by-case basis, which may lead to a vast variety in the exact APCs to be paid for some authors. Lastly, we utilized the highest APCs per journal, which sometimes have lower APCs for article types other than original articles, such as case reports. However, as most original research is published as original articles, we believe this most accurately reflects the status of authors. 

## CONCLUSION

OA publishing is common across cardiology and cardiac surgery journals with substantial APCs. Fees are prohibitive for unfunded and lesser-funded researchers in the absence of broader waiver considerations.

**Table:** 

Authors' roles & responsibilities	
DV	Substantial contributions to the conception or design of the work; or the acquisition, analysis, or interpretation of data for the work; drafting the work or revising it critically for important intellectual content; final approval of the version to be published
JGYL	Drafting the work or revising it critically for important intellectual content; final approval of the version to be published
MPBOS	Drafting the work or revising it critically for important intellectual content; final approval of the version to be published
EWE	Substantial contributions to the conception or design of the work; or the acquisition, analysis, or interpretation of data for the work; drafting the work or revising it critically for important intellectual content; final approval of the version to be published
